# Identification of mitophagy-related biomarkers in human osteoporosis based on a machine learning model

**DOI:** 10.3389/fphys.2023.1289976

**Published:** 2024-01-08

**Authors:** Yu Su, Gangying Yu, Dongchen Li, Yao Lu, Cheng Ren, Yibo Xu, Yanling Yang, Kun Zhang, Teng Ma, Zhong Li

**Affiliations:** ^1^ Honghui Hospital, Xi’an Jiaotong University, Xi’an, China; ^2^ Department of International Ward (Orthopedic), Hospital of Chengdu University of Traditional Chinese Medicine, Chengdu, China; ^3^ Basic Medical College of Yan’an University, Yan’an, China

**Keywords:** bioinformatics, osteoporosis, mitophagy, differentially expressed genes (DEGs), biomarker, protein-protein interaction (PPI)

## Abstract

**Background:** Osteoporosis (OP) is a chronic bone metabolic disease and a serious global public health problem. Several studies have shown that mitophagy plays an important role in bone metabolism disorders; however, its role in osteoporosis remains unclear.

**Methods:** The Gene Expression Omnibus (GEO) database was used to download GSE56815, a dataset containing low and high BMD, and differentially expressed genes (DEGs) were analyzed. Mitochondrial autophagy-related genes (MRG) were downloaded from the existing literature, and highly correlated MRG were screened by bioinformatics methods. The results from both were taken as differentially expressed (DE)-MRG, and Gene Ontology (GO) analysis and Kyoto Encyclopedia of Genes and Genomes (KEGG) enrichment analysis were performed. Protein-protein interaction network (PPI) analysis, support vector machine recursive feature elimination (SVM-RFE), and Boruta method were used to identify DE-MRG. A receiver operating characteristic curve (ROC) was drawn, a nomogram model was constructed to determine its diagnostic value, and a variety of bioinformatics methods were used to verify the relationship between these related genes and OP, including GO and KEGG analysis, IP pathway analysis, and single-sample Gene Set Enrichment Analysis (ssGSEA). In addition, a hub gene-related network was constructed and potential drugs for the treatment of OP were predicted. Finally, the specific genes were verified by real-time quantitative polymerase chain reaction (RT-qPCR).

**Results:** In total, 548 DEGs were identified in the GSE56815 dataset. The weighted gene co-expression network analysis(WGCNA) identified 2291 key module genes, and 91 DE-MRG were obtained by combining the two. The PPI network revealed that the target gene for AKT1 interacted with most proteins. Three MRG (NELFB, SFSWAP, and MAP3K3) were identified as hub genes, with areas under the curve (AUC) 0.75, 0.71, and 0.70, respectively. The nomogram model has high diagnostic value. GO and KEGG analysis showed that ribosome pathway and cellular ribosome pathway may be the pathways regulating the progression of OP. IPA showed that MAP3K3 was associated with six pathways, including GNRH Signaling. The ssGSEA indicated that NELFB was highly correlated with iDCs (cor = −0.390, *p* < 0.001). The regulatory network showed a complex relationship between miRNA, transcription factor(TF) and hub genes. In addition, 4 drugs such as vinclozolin were predicted to be potential therapeutic drugs for OP. In RT-qPCR verification, the hub gene NELFB was consistent with the results of bioinformatics analysis.

**Conclusion:** Mitophagy plays an important role in the development of osteoporosis. The identification of three mitophagy-related genes may contribute to the early diagnosis, mechanism research and treatment of OP.

## 1 Introduction

Osteoporosis (OP) is a systemic bone disease and the most common metabolic bone disease (Langdahl, 2020; Yan et al., 2023). They can be divided into two categories: primary and secondary. The former mainly occurs in children and young adults, considering the possible influence of genetic factors; however, its specific pathogenesis remains unknown. The latter is more common, mainly affecting older men and postmenopausal women, and is related to aging (Sozen et al., 2017; Guo et al., 2021). The main feature of OP is uncoupled bone resorption, which leads to decreased bone mass, damage, and degradation of the bone tissue microstructure, resulting in increased bone fragility and increased susceptibility to fractures, especially in the hip, wrist, and spine (Liu et al., 2021). OP is reported to be associated with fractures in more than half of white women and one-third of white men (Golob and Laya, 2015). Age is the most important risk factor of OP(6). In addition, estrogen deficiency, improper use of glucocorticoids, diabetes, malnutrition, and heavy drinking have been identified as important risk factors for OP(6, 7). The invisibility of bone loss and absence of symptoms at the beginning of OP make it a silent disease, as it is usually difficult to detect before the first fracture; however, damage can be fatal once it occurs (Unnanuntana et al., 2010; Johnston and Dagar, 2020).

Currently, the diagnostic methods for OP are based on Dual-Energy X-Ray Absorptiometry (DEXA) evaluation of bone mass (Chen et al., 2023), double X-ray absorption measurement (Aibar-Almazan et al., 2022), quantitative computed tomography, and bone tissue biopsy (Filippiadis et al., 2018). However, owing to its high detection cost, strong invasiveness, and large amounts of ionizing radiation, its long-term use is limited. There are three types of treatment options for OP, which are divided into anti-reabsorption therapy, anabolic therapy, and dual-acting therapy (Foessl et al., 2023). Anti-reabsorption therapy involves the use of anti-reabsorption drugs, including bisphosphonates, denosumab, selective estrogen receptor modulators (SERMs), and estrogen, to reduce bone resorption by inhibiting osteoclast function (Chen et al., 2023; Foessl et al., 2023). Anabolic drugs, such as teriparatide, which is used in anabolic therapy, is strong bone formation promoter effective in treating osteoporosis (Che et al., 2023). Romosozumab is currently the only drug available as dual-acting therapy. The dual effects of increased bone formation and reduced bone resorption lead to rapid increases in trabecular and cortical bone mass, which can improve bone structure and strength (Langdahl, 2020). However, most drugs have limitations and adverse side effects, and the current clinical treatments for OP are not ideal. Osteoclast-mediated bone destruction can occur, resulting in impaired therapeutic effects (Nogues and Martinez-Laguna, 2018). Therefore, there is an urgent need to develop new diagnostic methods for OP and provide new targets for the development of new drugs and immunotherapy by understanding the biological pathways of the markers.

Autophagy is a membrane-dependent, subcellular component turnover mechanism in eukaryotic cells. It is a complex dynamic process that maintains intracellular homeostasis under different physiological or pathological conditions by controlling protein degradation and the renewal of damaged organelles (Wang J. et al., 2023). Mammalian autophagy is divided into three subcategories: macroautophagy, microautophagy, and chaperone-mediated autophagy. Macroautophagy is the main autophagic process that helps effectively transport cytoplasmic cargo to lysosomes for degradation (Mizushima and Levine, 2020). Autophagy is highly regulated and an appropriate level of autophagy can protect cells from pathological or physiological damage. Conversely, excessive or reduced autophagy trigger apoptosis (Scheibye-Knudsen et al., 2014). Studies have shown that autophagy is necessary for cell survival. Dysregulation of autophagy is associated with various human diseases, including cancer and metabolic, neurodegenerative, cardiovascular, and age-related diseases (Mattson et al., 2008).

Mitochondrial autophagy is a selective form of autophagy. It is a autophagy process that occurs in mitochondria. This is a highly specific quality control process and the only known selective method for removing entire mitochondria. It maintains mitochondrial homeostasis by eliminating damaged organelles and redundant proteins, and reducing cell stress caused by harmful stimuli (Zong et al., 2016; Wang J. et al., 2023). Mitochondrial autophagy maintains cellular homeostasis under healthy conditions. However, it can also be induced under certain pathological or physiological conditions, thereby degrading healthy mitochondria and ultimately promoting the development of many diseases (Fang et al., 2019; Xu et al., 2020; Zhang et al., 2021). In addition, mitophagy has shown excellent medical application value in many chronic diseases and is considered to be an important target for the treatment of chronic diseases (Yan et al., 2023). Increasing evidence shows that abnormal mitochondrial autophagy can disrupt the balance of bone metabolism and plays a key role in bone metabolism disorders (Wang et al., 2017; Naik et al., 2019; Wang S. et al., 2020). Other studies have shown that mitophagy is involved in the regulation of OP. For example, phosphoinositide 3-kinase (PI3K) can treat or affect the progression of OP by interfering with mitophagy in bone marrow-derived mesenchymal stem/stromal cells (BMSC) (Yang et al., 2019; Chen et al., 2020). Lee found that the expression of Pten-induced putative kinase 1 (PINK1) was decreased in OP patients, and the PINK1/Parkinson‘s disease-related gene (Parkin) pathway is an important way of mitophagy (Lee et al., 2021). Maity et al. (2022) reported that the expression levels of mitophagy-related molecules PINK1 and Parkin in DPSC were increased, and mitophagy was induced to promote osteoblast differentiation. It has also been reported that epigallocatechin-3-gallate (EGCG) is a rich polyphenol in green tea, which can significantly reduce the expression of mitochondrial autophagy related PINK1 and Parkin. inhibit osteoclast differentiation, and regulae the progression of osteoporosis (Sarkar et al., 2022). All of the above prove that mitophagy may be related to OP. Therefore, we have reason to suspect that osteoporosis is associated with mitophagy. However, previous studies have only explored the effect of a specific gene-mediated mitophagy pathway on osteoporosis, suggesting that there is a certain correlation between the two. There is still a lack of research on the clinical significance of mitophagy-related gene (MRG) in osteoporosis. Therefore we conducted this study to understand the expression of mitophagy-related genes in OP, in order to provide an important reference for the diagnosis, mechanism research and treatment of OP.

## 2 Methods and materials

### 2.1 Data sources and downloaded data

The clinical data of all patients were obtained from the Gene Expression Omnibus (GEO) database (https://www.ncbi.nlm.nih.gov/geo/). According to the following criteria: (Yan et al., 2023) Disease name: Osteoporosis (Langdahl, 2020); Organism type: *Homo sapiens* (Guo et al., 2021). Sample source: blood sample (Guo et al., 2021). Dataset including samples with low and high bone mineral density (BMD) (Sozen et al., 2017). Sample size as large as possible (The dataset contains more than 3 samples per group). Hence, GSE56815, including the gene expression data (Expression profiling by array) of peripheral blood monocytes from 40 low-BMD samples and 40 high-BMD samples, was analyzed in this study. Thirty-four mitophagy-related genes (MRG) were downloaded from previous literature (Xu et al., 2022).

### 2.2 Identification of differentially expressed genes

We used the Wilcoxon test method in the R package to analyze the genetic data and get individual *p*-values after we completed the data download and collation (Cheng L. et al., 2022). The false discovery rate (FDR) was used to test the *p*-value, and the FDR value obtained was used to screen the differentially expressed genes (DEGs), with the following criterion:FDR<0.05 (Hsu and Lee, 2010). Volcano plots and heat maps were generated to identify the DEGs.

### 2.3 Screening of module genes related to mitophagy

In the GSE56815 dataset, the expression matrices of 34 MRG were extracted, and the MRG scores between OP and control samples were calculated using the gene set variation analysis (GSVA) package (version 1.42.0) (Suarez-Farinas et al., 2010). The weighted gene co-expression network analysis (WGCNA) package (version 4.0.3) (Langfelder and Horvath, 2008) in R was used to screen out module genes with high correlation with MRG score. Specifically, samples were clustered and outliers were excluded to ensure the accuracy of the analysis, and the appropriate soft threshold was determined with the following conventional criterion: 0.8 < R^2^ < 0.95, and the mean value of the adjacency function was close to 0. A co-expression matrix was constructed, and a hybrid dynamic clipping tree method with a minimum of 30 modules was used to identify co-expressed gene modules (Reyes et al., 2017), and the huge number of genes was classified into dozens of gene modules. Finally, the significant modules correlated with MRG scores were defined with a relevance |Cor|>0.3, *p* < 0.05.

### 2.4 Gene Ontology and Kyoto Encyclopedia of Genes and Genomes enrichment analysis of differentially expressed-MRG

The online jvenn website (http://jvenn.toulouse.sra.fr/app/example.html) was used to intersect the module MRG screened by DEGs and WGCNA to obtain differentially expressed-MRG, which were denoted as DE-MRG. The R package “clusterProfiler” (version 4.4.4) (Yu et al., 2012) was used to perform Gene Ontology (GO) and Kyoto Encyclopedia of Genes and Genomes (KEGG) enrichment analysis of DE-MRG. A *p*-value <0.05 was considered statistically significant. The R package “ggplot2” (version 3.3.2) (Ito and Murphy, 2013) was used to visualize the enrichment results.

As part of the GO analysis, three ontologies were identified, namely molecular function (MF), cellular component (CC), and biological process (BP), which helped to explore the biological processes of these DEGs. KEGG analysis was used to identify metabolic or signal transduction pathways that were significantly enriched in the DEGs.

### 2.5 Specific protein-protein interaction network construction

At present, protein-protein interaction (PPI) networks are among the best-studied biomolecular networks. To explore the interaction between DE-MRG, we used the STRING ( https://string-db.org ) website to construct the PPI network of DE-MRG and screen the threshold: confidence = 0.4 (Zhao et al., 2015), and then visualized with Cytoscape (Shannon et al., 2003).

### 2.6 Signature gene identification

We identified candidate key genes by the intersection of key module genes and DGEs. Subsequently, three machine learning algorithms, least absolute shrinkage and selection operator (Lasso), support vector machine recursive feature elimination (SVM-RFE), and Boruta were used to analyze the hub genes.

Lasso regression is a new variable selection technique, which is implemented by using the glmnet package with penalty parameters, and the feature genes are obtained after 10-fold cross validation. SVM-RFE is an effective feature selection technique using the “e1071” package and “glmnet” package (Friedman et al., 2010) to obtain the relationship between the prediction accuracy and the number of features, and the number of hub genes is obtained when the generalization error is the lowest. The Boruta algorithm is a wrapper method built around a random forest classifier (Shen et al., 2021). This algorithm minimizes the error of the random forest model, which eventually forms a subset of the minimum optimal features.

Using the online Jveen map-making website (http://jvenn.toulouse.inra.fr/app/example.html), overlapping genes were obtained by intersecting the markers derived from the three algorithms, that is, the key genes obtained by screening.

The receiver operating characteristic (ROC) curve of the hub genes was drawn using the R pROC package (version 1.12.1) (Hanley and McNeil, 1982) and the diagnostic efficiency of the key genes was evaluated using the area under the curve (AUC). An AUC greater than 0.7 indicated good diagnostic performance.

The nomogram model can evaluate the relationship between variables in the prediction model (Park, 2018). The nomogram model of the hub genes was constructed using the R RMS package (version 6.0-1) (Zhao and Li, 2023), and a calibration curve was drawn using the calibration function and boot method. The accuracy of the nomogram model for predicting osteoporosis was determined using the slope of the curve and Hosmer-Lemeshow goodness-of-fit test (HL test), where the insignificant difference (*p* > 0.05) between predicted and actual observed results in HL test indicates that the prediction model has good calibration ability (Wang et al., 2021).

### 2.7 Gene Ontology and Kyoto Encyclopedia of Genes and Genomes enrichment analysis of hub genes

The Pearson correlation coefficient between the key genes and the remaining genes was calculated and ranked from highest to lowest. After the corresponding gene sequence list of each key gene was obtained, the “clusterProfiler” package (version 4.4.4) (Yu et al., 2012) was used for GO and KEGG Gene Set Enrichment Analysis (GSEA), and Benjamini & Hochberg method was used for multiple test correction. The corrected *p*-value was p.adjust, and p.adjust <0.05 was considered as significant enrichment.

### 2.8 Classical pathways of the DEGs were analyzed by IPA pathway analysis

IPA Pathway Analysis (IPA) is an integrated analysis software. We used DEGs to perform IPA functional enrichment analysis to identify typical pathways, diseases, and functional pathways related to key genes. A z-score >2 indicates that the pathway is activated, and a z-score < -2 indicates that the pathway is inhibited. All pathways containing key OP genes were screened for display.

### 2.9 Immune microenvironment analysis

Single sample GSEA (ssGSEA) was performed by calculating the rank value of each gene based on the expression profile. To explore the composition of immune cells in the GSE56815 dataset, ssGSEA package (version 1.36.3) (Zuo et al., 2020) was used to perform immune cell infiltration analysis, and the differentially expressed immune cells between the disease group and the control group were detected using Wilcoxon test (*p* value less than 0.05 was considered considerable). The results were presented as boxplots. Subsequently, the “spearman” algorithm was used to calculate the correlation between the immune-related gene sets and the correlation between OP hub genes and the significantly different immune-related gene sets, respectively. The results were presented as heat-and-bubble maps.

### 2.10 Network construction based on hub gene

#### 2.10.1 Construction of the ceRNA network

For the obtained OP hub genes, we used the miRWalk database (http://mirwalk.umm.uni-heidelberg.de/search_genes/) and miRTarBase database (https://mirtarbase.cuhk.edu.cn/∼miRTarBase/miRTarBase_2022/php/search.php) to search for relevant miRNAs, then took the intersection to obtain the miRNA of OP hub genes. Based on the obtained miRNAs, the databases ENCORI (https://starbase.sysu.edu.cn/agoClipRNA.php?source=mRNA) (the default parameters are set: CLIP-Data ≥ 1, Degradome-Data ≥ 0, pan-Cancer ≥ 0) and LncBase (https://diana.e-ce.uth.gr/lncbasev3/interactions) were searched; then, the corresponding lncRNAs of the miRNA obtained in the two databases were intersected to obtain the lncRNA of OP hub genes. Cytoscape (Shannon et al., 2003) was used to draw the visualized mRNA-miRNA-lncRNA network diagram.

#### 2.10.2 Construction of TF-hub gene network and predicting potential drugs

Based on the obtained key OP genes, NetworkAnalyst (https://www.networkanalyst.ca/NetworkAnalyst/uploads/ListUploadView.xhtml) was used to search for the TF corresponding to the key genes to obtain the interaction information between them, and parameter settings are as follows: Specify organism = “*H. sapiens* (human)”, Set ID type = “Official Gene Symbol” Cytoscape (Shannon et al., 2003) was used to visualize the interactions.

### 2.11 Predicting potential drugs

Potential drugs for the treatment of OP were predicted using the CTD database (http://ctdbase.org/), and the interactions between the two were analyzed. Cytoscape (Shannon et al., 2003) was used for the visual display.

### 2.12 Validation of key gene expression

From the GSE56815 dataset, the expression levels of key OP genes were extracted and analyzed using the Wilcoxon test. The expression of key genes was checked and the results are displayed as box plots.

### 2.13 Experimental validation by RNA extraction and real-time quantitative polymerase chain reaction

The study was in accordance with the Declaration of Helsinki (as revised in 2013) (World Medical Association, 2013). This study was approved by the Ethics Committee of Honghui Hospital Affiliated to Medical College of Xi ‘an Jiaotong University (No.202212012). All participants were informed and signed consent. The study included patients who met the criteria in the orthopedics department of the hospital from January 2023 to November 2023.

Inclusion criteria: (Yan et al., 2023) Those who met the diagnostic criteria of OP, according to “Chinese expert consensus on the diagnosis of osteoporosis by imaging and bone mineral density” (Cheng et al., 2020; Langdahl, 2020). All subjects were informed and voluntarily signed the consent form.

Exclusion criteria (Yan et al., 2023): those who do not meet the diagnosis of OP; (Langdahl, 2020) patients with severe cardiovascular disease (Guo et al., 2021); Patients with severe liver/kidney dysfunction (Sozen et al., 2017); patients with coagulation dysfunction (Liu et al., 2021); patients with autoimmune diseases (Golob and Laya, 2015); untreated bone metabolic diseases other than osteoporosis (Johnston and Dagar, 2020); Long-term use of drugs affecting bone metabolism (such as glucocorticoids and steroid hormones) (Unnanuntana et al., 2010); Anti-osteoporosis treatment history (such as bisphosphonates, teriparatide and calcitonin, etc.).

Grouping criteria: lumbar spine and total hip BMD (DEXA): normal group T-score≥1.0; T-score ≤ -2.5 in OP group (all BMD measurements were performed by the same trained professional).

Finally, 8 patients with osteoporosis and 8 patients with normal bone mineral density were included in the study. There were 4 males and 4 females in each group. Peripheral venous blood (10 mL) was collected from all patients after fasting for one night (≥12 h).

The operation of human peripheral blood mononuclear cells (PBMC) is carried out as described above (Chen et al., 2021). First, the patient‘s whole blood was placed in a 50 mL centrifuge tube, diluted with 10 mL PBS and gently mixed. Subsequently, the blood sample was continuously centrifuged for 20 min at a speed of 2000 rpm. After centrifugation, the white blood cell layer in the center of the sample containing PBMC was sucked out with a pipette and transferred to a new 15 mL centrifuge tube, and 10 mL PBS was added to it. The centrifuge was used to continuously centrifuge for 10 min at a speed of 1500 rpm, and then the supernatant was taken out for precipitation, which was the required PBMC. The obtained PBMC were inoculated in a 6-well plate, and 1 mL of TRIzol (GENSTAR Inc. Beijing, China) reagent was added to each well to extract total RNA from the cells. Subsequently, 1 mg total RNA was reverse transcribed using a cDNA synthesis kit (Thermo Fisher Scientific Inc. Shanghai, China). The cDNA was detected using a 20 mL SYBR Green qPCR SuperMix (TargetMol Chemicals Inc. Shanghai, China) and RT-qPCR machine (Bio-Rad, Hercules, CA, United States). The final thermal cycle conditions for gene amplification were: 95 C 30 s, 95 C 5 s 40 cycles, and the last step was 60 C 30 s. Quantitative analysis was performed using the 2^△△CT^ method to calculate the relative expression of each gene. The hub gene-related detection primers were prepared by Beijing Tsingke Biotech Co., Ltd. (China), as shown in Table 1.

**TABLE 1:** Sequences of hub gene-specific primers used for RT-qPCR.

Hub genes	Sequence (5′->3′)	
	Forward primer	Reverse primer
NELFB	GGC​TGT​ACT​ACG​TCC​TGC​ACA​T	AGG​TGG​AGG​AAG​ATG​TCG​CCA​A
SFSWAP	ACG​CTA​CAC​TGT​CCT​GGC​AGA​A	AGG​CGG​TTA​CAC​TTC​TTG​GAG​G
MAP3K3	CCA​GTT​GAA​GGC​TTA​CGG​TGC​T	AGA​GTC​TCG​GAG​GAT​GTT​GGC​T

### 2.14 Statistical analysis

All statistical analyses were performed using R software. Differentially expressed analysis for DEGs, differentially expressed immune cells, and key OP genes between OP and control samples in GSE56815 datasets were conducted using Wilcoxon test. When *p* < 0.05, all results were considered statistically significant and all *p*-values were two-tailed.

**FIGURE 1:**
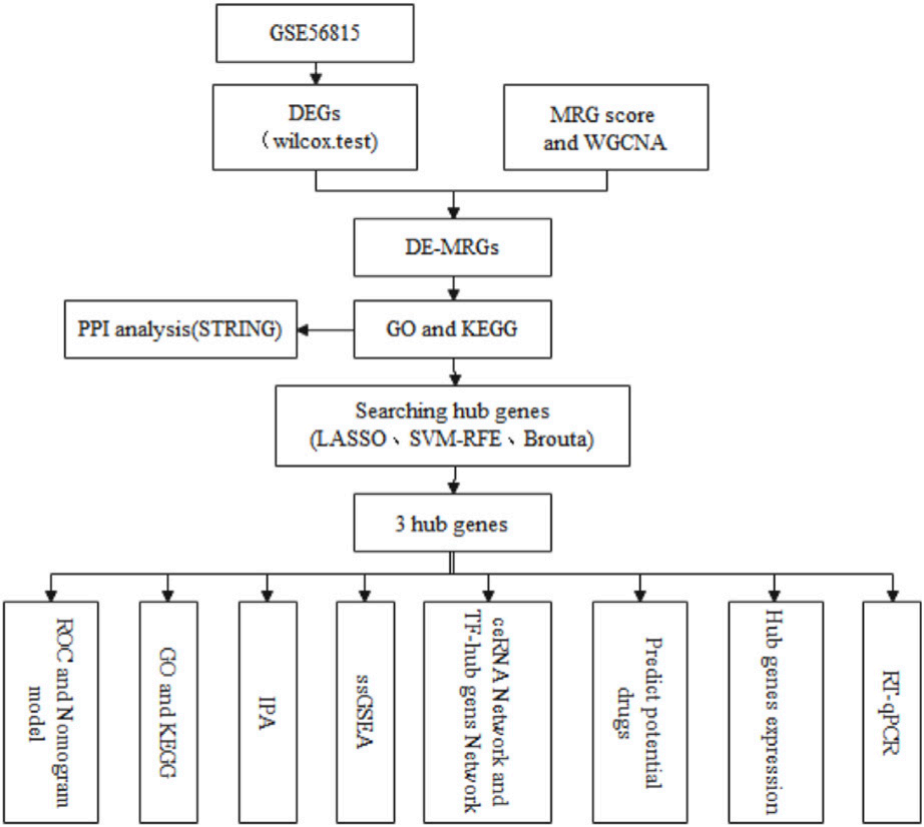
The flow chart of this research.

The flow chart of this study is shown in Figure 1.

## 3 Results

### 3.1 Differential gene analysis

**FIGURE 2:**
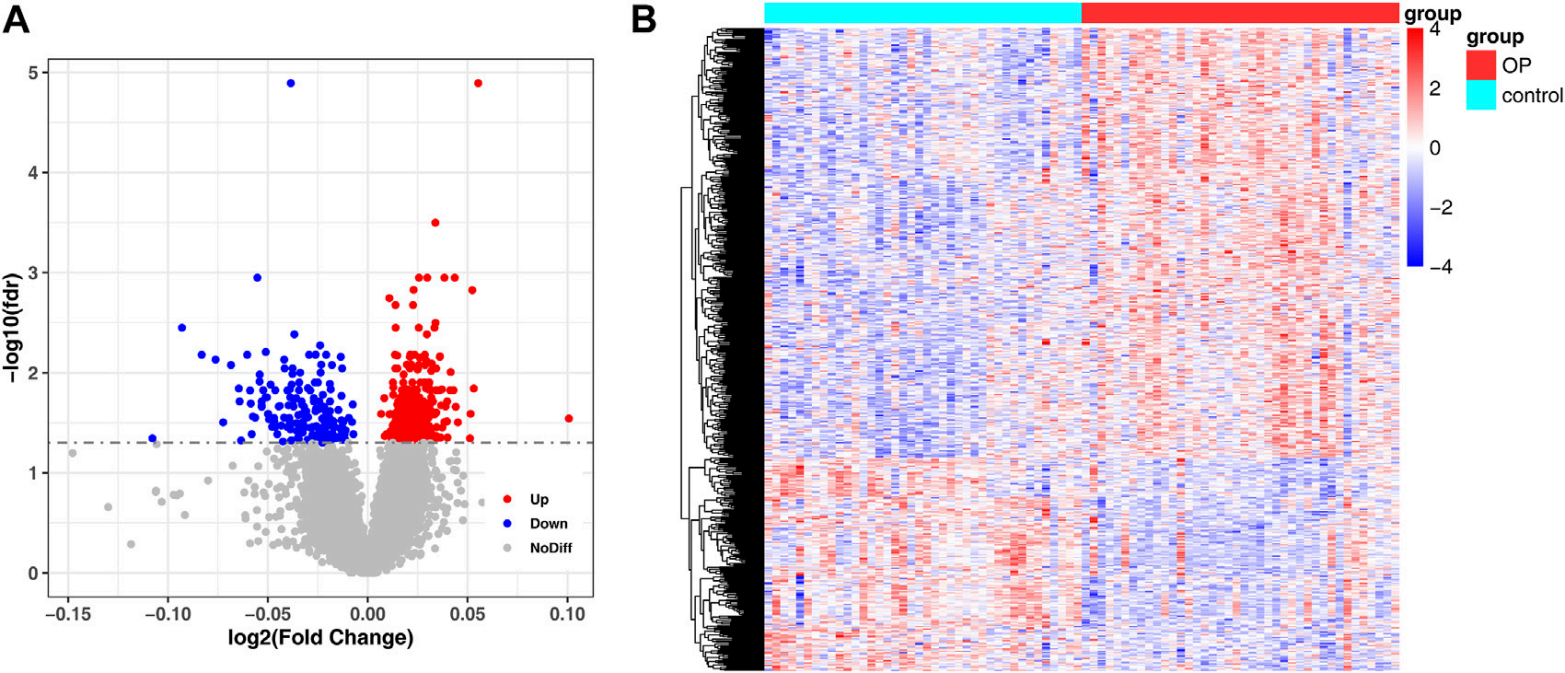
Detection of DEG between low and high BMD samples. **(A)** Volcano plot of differentially expressed genes identified in GSE56815 dataset. **(B)** Heat map of differentially expressed genes identified in GSE56815 dataset.

In the GSE56815 dataset, 548 genes were detected between low- and high-BMD samples, of which 366 genes were upregulated and 182 genes were downregulated (Figures 2A, B; Supplementary Table S1).

### 3.2 GSVA and WGCNA screen mitochondrial autophagy-related genes scoring related modules

The expression matrix of the 34 MRG was obtained from the dataset GSE56815. MRG scores were calculated between OP samples and control samples using the “GSVA” package.

**FIGURE 3:**
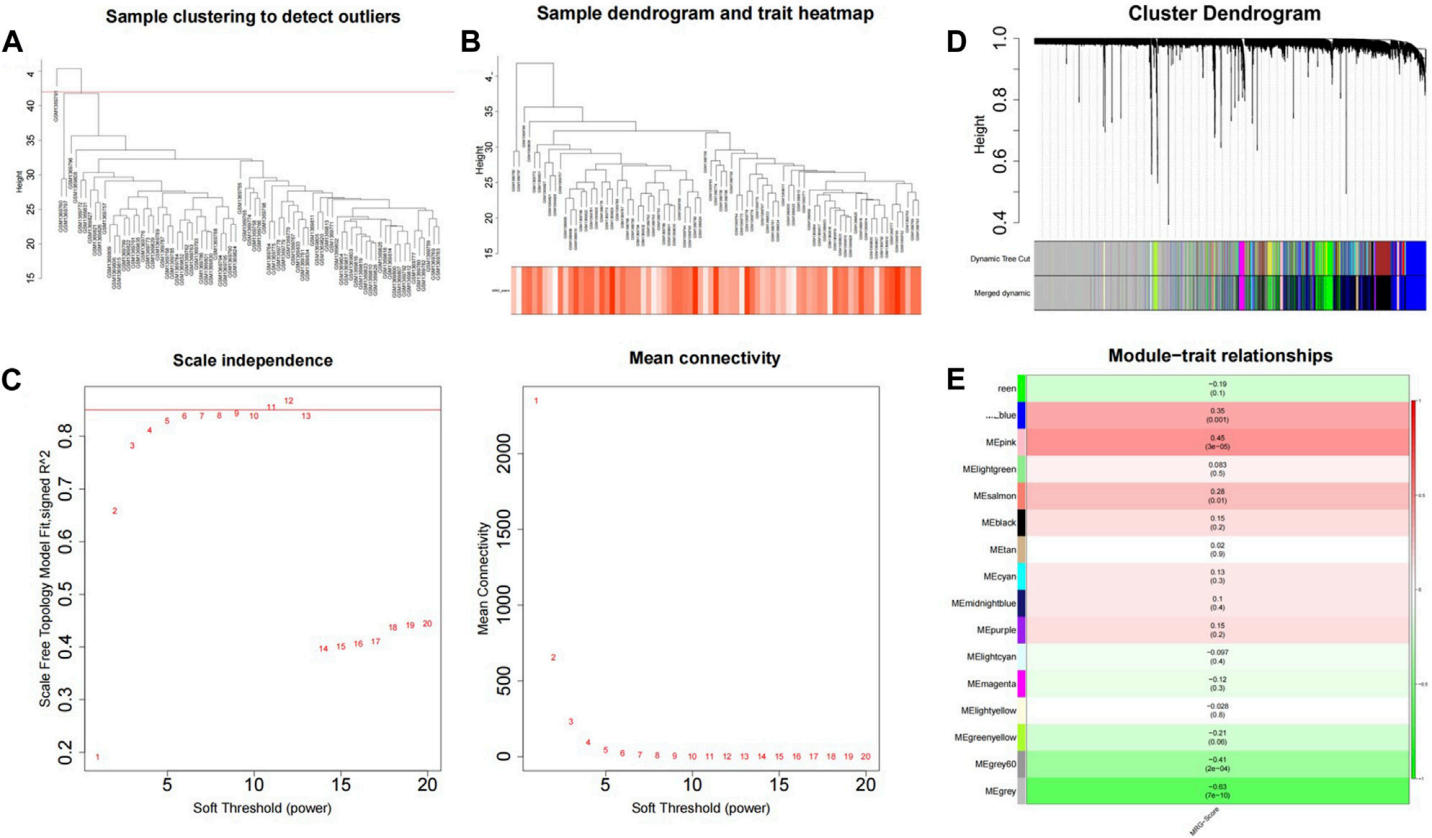
GSVA combined with WGCNA was used to screen MRG score related modules. **(A)** Sample clustering tree. **(B)** Data sample re-clustering and phenotypic information. **(C)** The soft threshold distribution. **(D)** The cluster dendregram after module merging. **(E)** The clustered modules.

The WGCNA package was used to analyze GSE56815, and the MRG score was used as WGCNA trait data to screen out module genes with high correlation with the MRG score; the samples were clustered to show the overall correlation of the samples in the data set. The threshold was set to 42 and outlier samples were eliminated, that is, those above the red line in the figure (Figure 3A). It was then reclustered, and the corresponding sample traits were added (Figure 3B). When β = 11, the co-expression network most closely approximates the scale-free distribution (Figure 3C). When MEDissThres = 0.3 was set, similar modules were analyzed, and the number of modules that aggregated after merging was 16 (Figure 3D). Analysis of the correlation of each module and the MRG score of red represents MRG scores are related, green represents is negatively related to the MRG score, color represents the degree of correlation, in | Cor | > 0.3, *p* < 0.05, for threshold selection, obtain results with MRG score high correlation modules with a total of three, blue (number of genes:1947), pink (number of genes:278), grey60 (number of genes:66), and the total number of genes was 2291(Figure 3E).

### 3.3 Identification and functional enrichment of differentially-expressed mitochondrial autophagy-related genes

**FIGURE 4:**
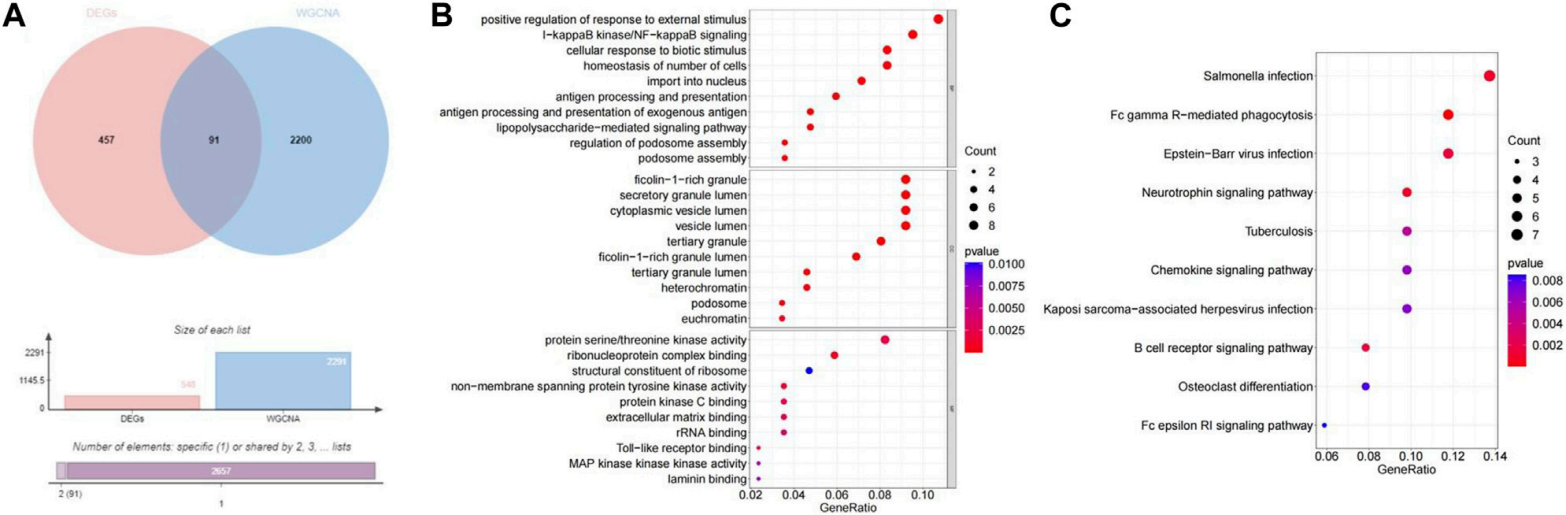
Identification and functional enrichment of DE-MRGs. **(A)** The veen plot showed the interaction between DEGs and MRGs. **(B)** The top 10 functional enrichment in BP, CC, and MF analysis, respectively.**(C)** The KEGG of DE-MRGs.

The 2291 hub module genes obtained by WGCNA overlapped with 548 DEGs obtained by differential analysis between the high- and low-BMD groups, and a total of 91 overlapping genes were obtained, namely, DE-MRG (Figure 4A). DE-MRG were subjected to GO functional enrichment and KEGG pathway analysis and were enriched in 430 biological processes (BPs), 59 molecular functions (MFs), 40 cellular components (CCs) (Figure 4B), and 33 pathways (Figure 4C) (*p* < 0.05). For BPs, the top 10 enrichment programs were mainly involved in the positive regulation of response to external stimuli, I-kappaB kinase/NF-kappaB signaling, cellular response to biotic stimulus, homeostasis of number of cells, import into nucleus, and other biological processes. For CC and MF, the top 10 enriched items are shown in Figures, respectively, and the enrichment analysis of KEGG pathway showed that DE-MRG are primarily involved in *Salmonella* infection, Fc gamma R-mediated phagocytosis, Epstein-Barr virus infection, and neurotrophin signaling pathways.

### 3.4 Protein-protein interaction analysis

**FIGURE 5:**
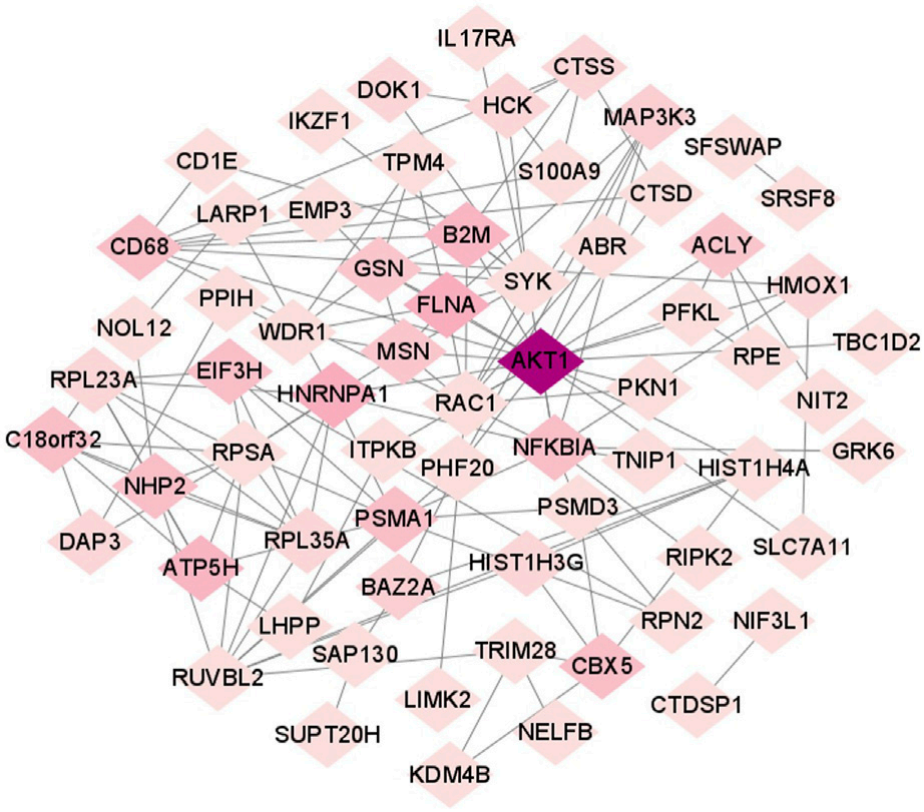
OP-specific protein-protein interaction network.

The construction of PPI network showed that gene AKT1 was the central gene of this PPI network, and it interacted with the most proteins. The results were visualized using the Cytoscape software (Figure 5).

### 3.5 Screening for the best characteristic genes of osteoporosis and evaluating their diagnostic value

**FIGURE 6:**
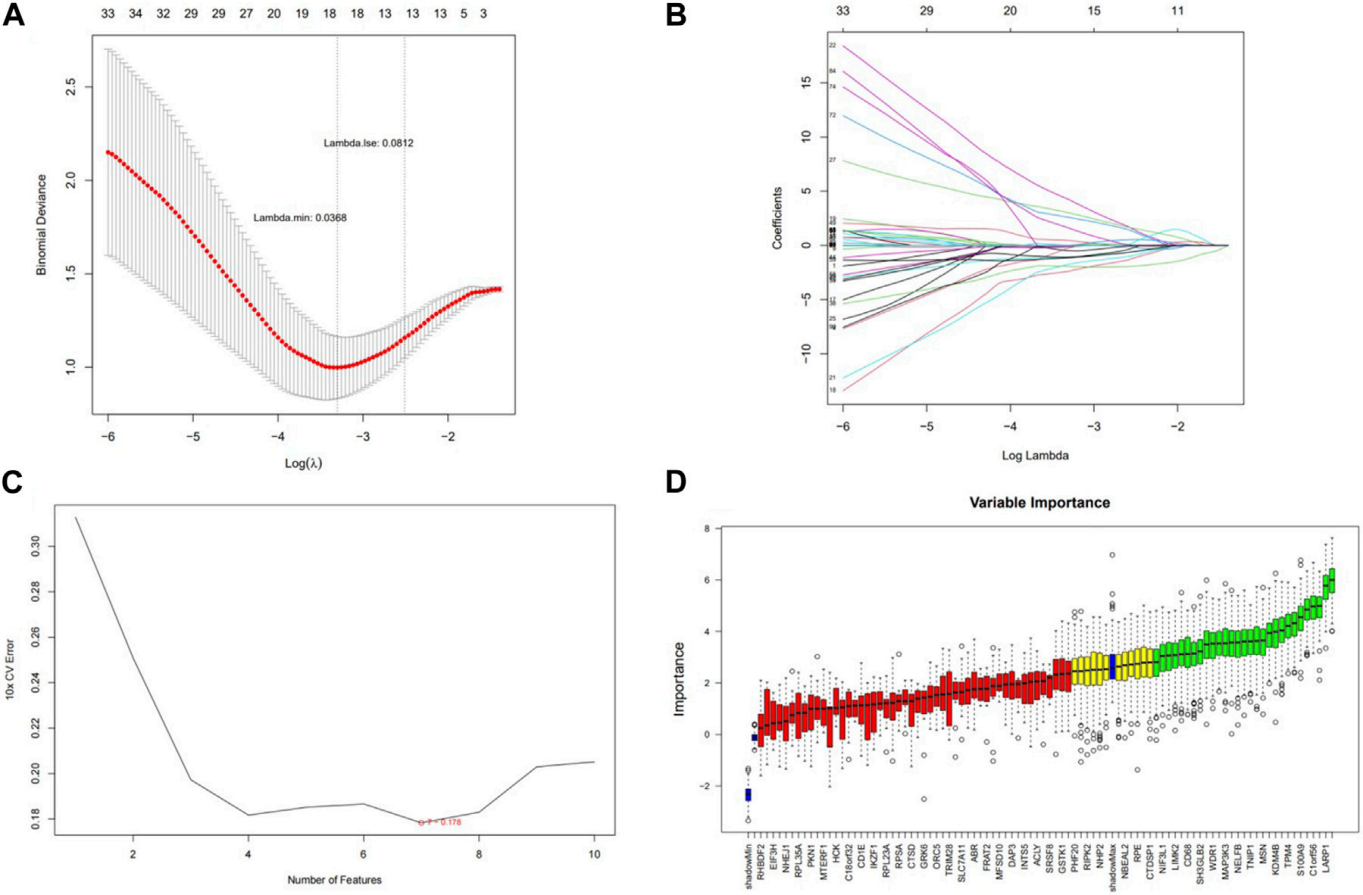
Screening hub genes and evaluating their diagnostic value. **(A)** LASSO logic coefficient penalty diagram. **(B)** LASSO plot showed the variations in the size of coefficients for parameters shrank as the value of k penalty increased. **(C)** The relationship between the prediction accuracy of SVM-RFE and the number of features. **(D)** Boxplot of importance distribution of each gene in Boruta algorithm.

**FIGURE 7:**
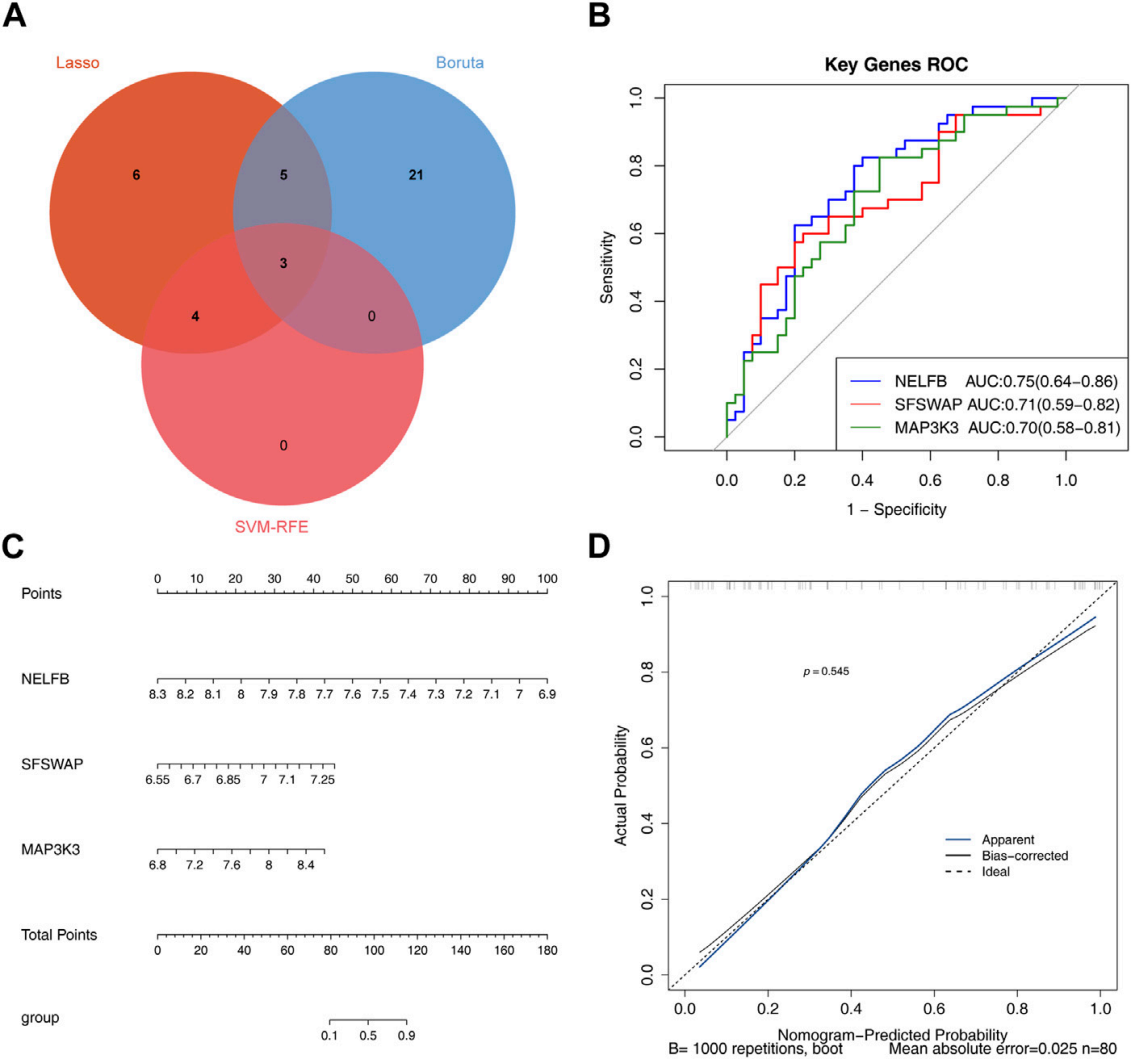
Evaluating the diagnostic value of hub genes **(A)** The veen plot showed the interaction of the LASSO, SVM-RFE and Boruta. **(B)** Receiver operating characteristic curve of three hub genes between OP group and control group. **(C)** nomogram model. **(D)** Calibration curve to evaluate the predictive ability of nomogram model.

A total of 18, 7, and 28 genetic biomarkers were identified using the machine learning algorithms LASSO, SVM-RFE, and Boruta, respectively (Figures 6A–D). Moreover, the key genes shared by the three OP were identified by overlapping biomarkers derived from the three algorithms, including negative elongation factor complex member B (NELFB), Splicing Factor (SFSWAP), and mitogen-activated protein kinase kinase 3 (MAP3K3) (Figure 7A).

We determined the reliability of the diagnostic value of our hub genes by plotting a ROC curve and calculating the area under the AUC; those of NELFB, SFSWAP, and MAP3K3 were 0.75(95% CI = 0.64-0.86), 0.71(95% CI = 0.59-0.82), and 0.70(95% CI = 0.58-0.81), respectively, with all greater than 0.7 (Figure 7B). Therefore, our gene hub can effectively diagnose osteoporosis. After the construction of the nomogram model of the gene hub(C-index = 0.886, 95% CI = 0.815-0.957), the slope of the calibration curve was close to 1, and the HL test exhibited satisfactory agreement between predicted and actual outcomes with *p* = 0.545 > 0.05, indicating that the nomogram model had high prediction accuracy for osteoporosis (Figures 7C, D).

### 3.6 Functional enrichment analysis of hub genes

**FIGURE 8:**
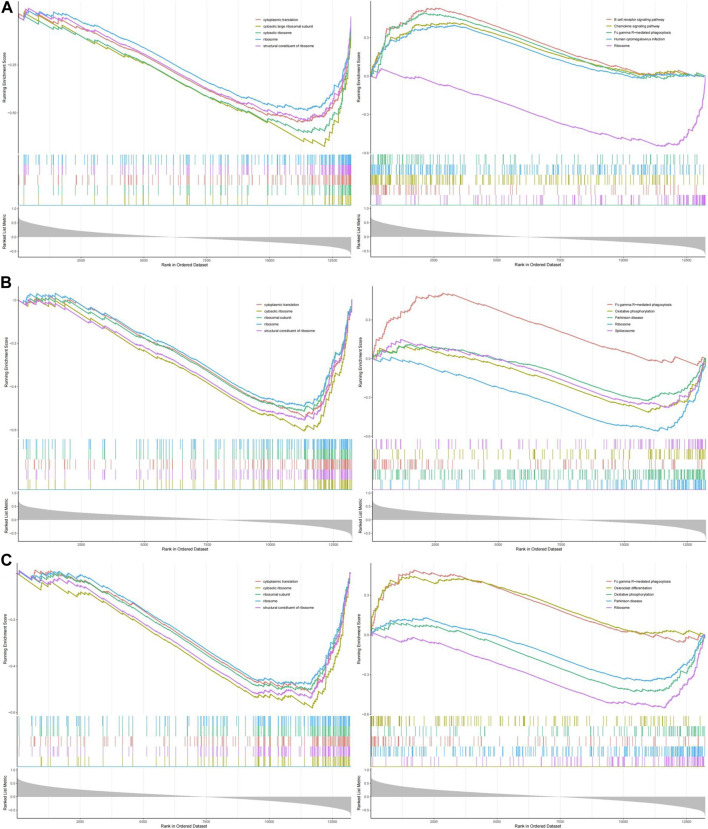
The GESA of OP hub genes. **(A)** The GSEA of NELFB in OP. **(B)** The GSEA of SFSWAP in OP. **(C)** The GSEA of MAP3K3 in OP.

GSEA enrichment analysis of GO and KEGG was performed on the three hub genes, and p.adjust <0.05 was considered a significant enrichment result (Supplementary Table S2). According to the significance ranking, GO and KEGG TOP5 Descriptions were selected for visual display (Figures 8A–C).

We evaluated the signaling pathways associated with the key genes using GSEA. The first five signaling pathways are shown in the figure. GO analysis showed that NELFB was significantly associated with cytoplasmic translation, cytosolic, large ribosomal subunit, cytosolic ribosome, ribosome, and structural constituents of the ribosomes. SFSWAP expression was significantly correlated with cytoplasmic translation, cytosolic ribosomes, ribosomal subunits, ribosomes, and the structural constituents of ribosomes. MAP3K3 expression significantly correlated with cytoplasmic translation, cytosolic ribosomes, ribosomal subunits, ribosomes, and structural constituents of ribosomes.

KEGG results showed that NELFB was significantly correlated with the B cell receptor signaling pathway, chemokine signaling pathway, Fc gamma R-mediated phagocytosis, human cytomegalovirus infection, and ribosomes. Sfswap expression was significantly associated with Fc gamma R-mediated phagocytosis, oxidative phosphorylation, Parkinson’s disease, ribosomes, and spliceosomes. The expression of MAP3K3 was significantly associated with Fc gamma R-mediated phagocytosis, osteoclast differentiation, oxidative phosphorylation, Parkinson’s disease, and ribosomes.

In summary, GO analysis showed that the molecular functions of these genes were mainly enriched with cytoplasmic translation, cytosolic ribosome, ribosome and structural constituent of ribosome; KEGG pathway analysis showed that it was mainly enriched in Fc gamma R-mediated phagocytosis and Ribosome.

### 3.7 IPA pathway analysis

**FIGURE 9:**
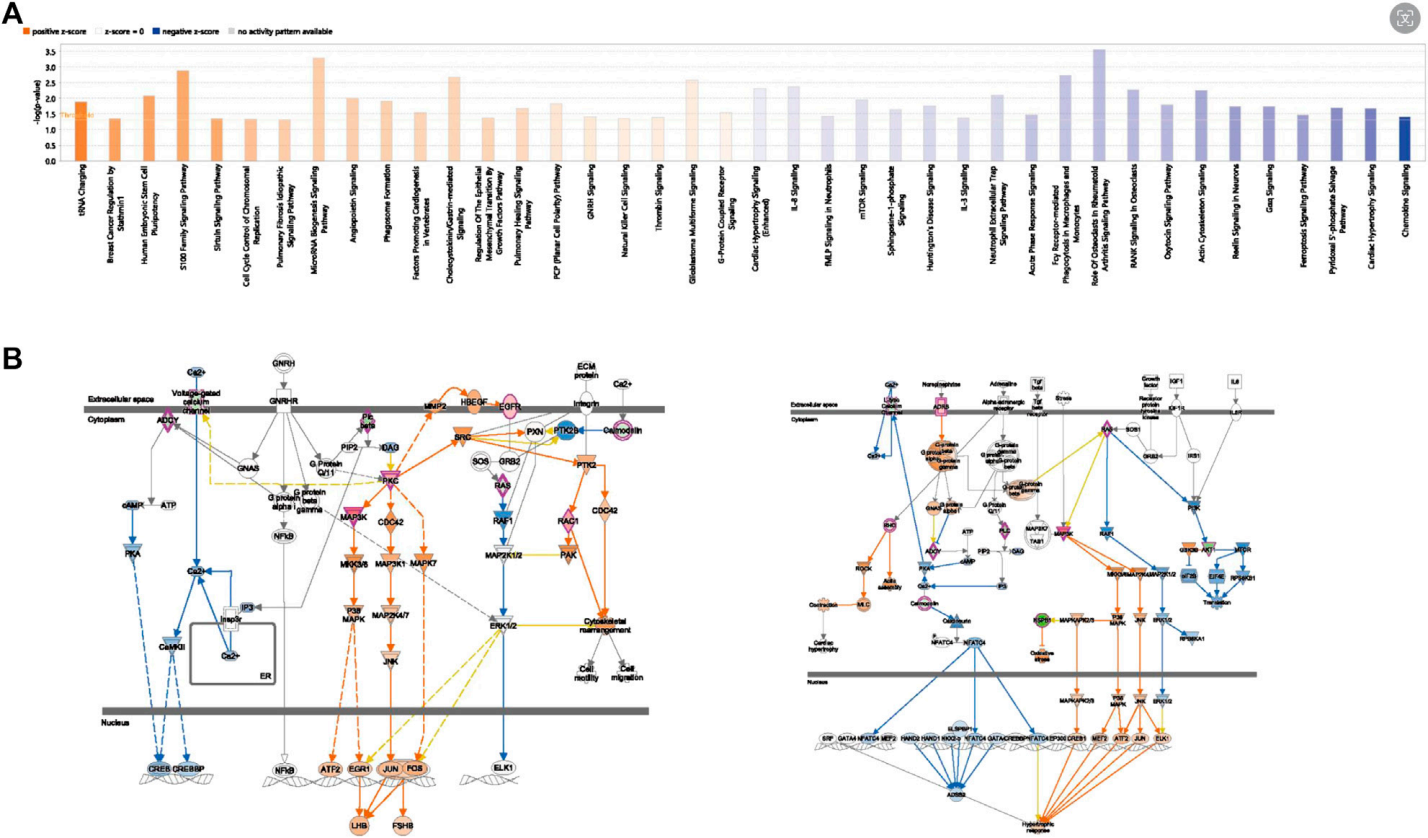
The IPA Pathway Analysis of OP hub genes. **(A)** Hub gene-related diseases and functional enrichment results. **(B)** MAP3K3 activation inhibition pathway diagram (left is the activation pathway, right is the inhibition pathway).

The results of the IPA functional enrichment analysis showed 20 inhibition pathways (blue) and activation pathways (range) (Figure 9A). The hub gene, MAP3K3, was included in these 40 pathways. The genes were involved in six pathways, and three activation pathways were GNRH Signaling, Natural Killer Cell Signaling and G-Protein Coupled Receptor Signaling. The three inhibited pathways were Cardiac Hypertrophy Signaling (enhanced), RANK Signaling in Osteoclasts, and Cardiac Hypertrophy Signaling. The strongest activation intensity of MAP3K3 (GNRH Signaling) and strongest inhibition intensity of the pathway (cardiac hypertrophy Signaling) are shown in Figure 9B.

### 3.8 Immune infiltration analysis of the dataset

**FIGURE 10:**
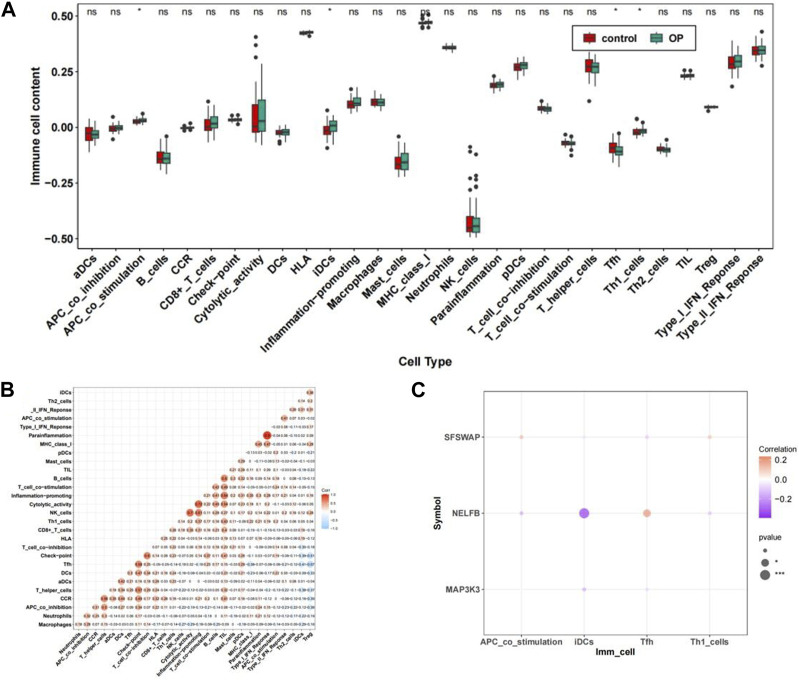
The immune cell infiltration association with hub genes. **(A)** Abundance box plots of 29 immune gene sets in OP group and Control group. **(B)** Heat map of the relationship between immune-related gene sets. **(C)** Bubble plot of correlation between OP hub genes and significantly different immune-related gene sets.**p < 0.01,***p < 0.001.

Immune infiltration analysis was performed on the integrated GSE56815dataset. The scores of the 29 immune cell types evaluated using ssGSEA showed significant differences in the expression of four immune-related gene sets: APC_co_stimulation, iDCs, Tfh, and Th1_cells (Figures 10A, B) (Supplementary Table S3). The correlations results between immune-related gene sets are shown in Figure 10B. (Supplementary Table S4). Further, the heatmap for the correlation of hub genes and immune-related gene sets suggested that there was a significant negative correlation between NELFB and iDCs in hub genes with significant differences (cor = −0.390, *p* < 0.001). There was a positive correlation with Tfh levels (cor = 0.233, *p* < 0.05) (Figure 10C).

### 3.9 Network construction based on hub genes

**FIGURE 11:**
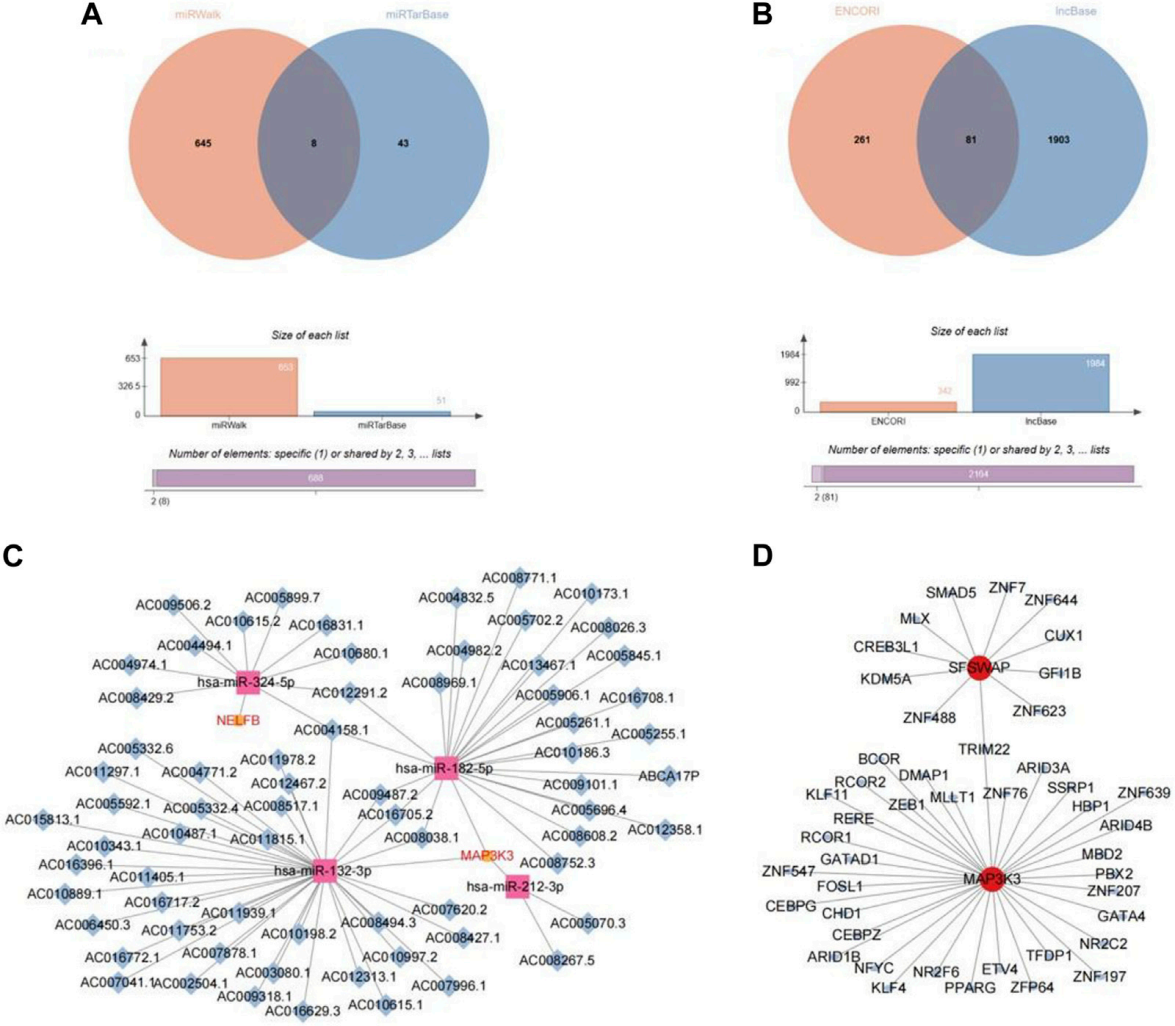
Network construction based on hub gene. **(A)** The veen plot of miRNA intersection predicted by miRwalk database and miRTarBase database. **(B)** The veen plot of lncRNA intersection predicted by ENCORI database and lncBase database. **(C)** CeRNA network diagram. **(D)** TF-mRNA network diagram.

We identified eight miRNAs, of which seven corresponded to MAP3K3 and one to NELFB (Figure 11A). Simultaneously, 81 lncRNAs were obtained, corresponding to four miRNAs (hsa-miR-132-3phsa-miR-182-5p, hsa-miR-212-3p, and hsa-miR-324-5p), which correspond to the key genes MAP3K3 and NELFB (Figure 11B). Based on the above two intersection pairs, 79 pairs of lncRNA-miRNA-mRNA relationships regulated by the same miRNA were screened, including 68 lncRNAs, four miRNAs, and two mRNAs, and a ceRNA network was constructed, that is, ceRNA network (Figure 11C).

In addition, 72 TF–mRNA interaction pairs were retrieved, and only the key genes, SFSWAP and MAP3K3, provided TF interaction information. Visual expression was performed using Cytoscape software (Figure 11D).

### 3.10 Potential drugs prediction

**FIGURE 12:**
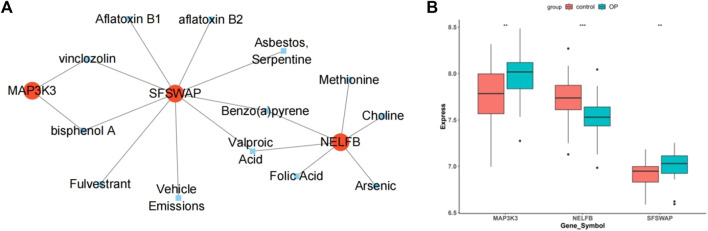
Drug prediction and gene expression levels. **(A)** Interaction network diagram of hub genes and potential drugs. **(B)** Hub genes in the data set GSE56815 expression box plot.

By predicting potential drugs for the treatment of OP in the CTD database, 17 drug-gene pairs were obtained. Both vinclozolin and bisphenol A are associated with SFSWAP and MAP3K3. Benzo (a) pyrene and valproic acid were associated with both SFSWAP and NELFB. The results were visualized using Cytoscape software (Figure 12A).

### 3.11 Dataset validation

The box plot shows that in the dataset GSE56815, compared with the control group, the genes MAP3K3 and SFSWAP were significantly expressed (*p* < 0.01), whereas the expression of NELFB was lower (Figure 12B).

### 3.12 RT-qPCR verification of hub genes

**FIGURE 13:**
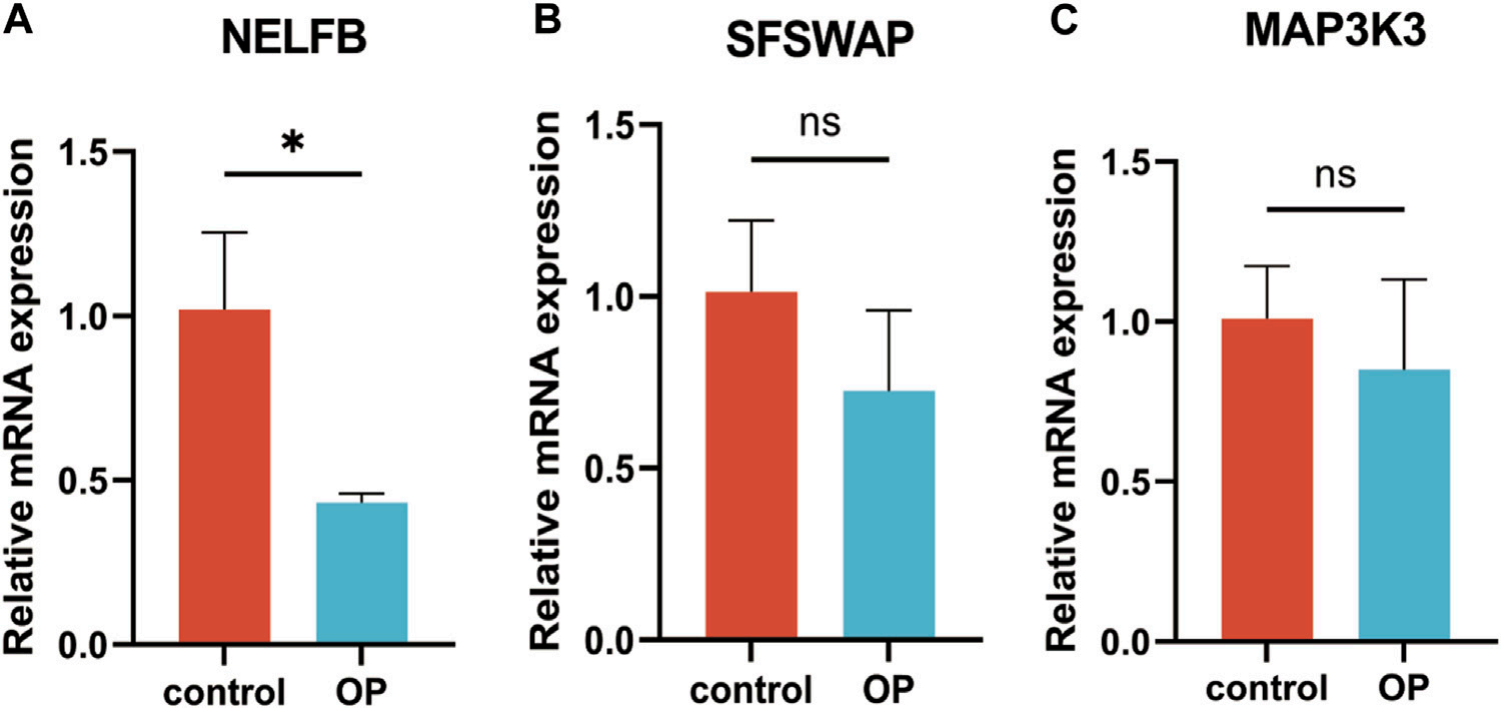
RT-qPCR verification of hub genes. **(A)** NELFB. **(B)** SFSWAP. **(C)** MAP3K3. All results were expressed as mean ± standard deviation.(*p < 0.05; ns, not significant).

The results of RT-qPCR showed that the mRNA expression level of NELFB in OP group was lower than that in normal group. There was no difference in the mRNA expression levels of SFSWAP and MAP3K3 between the OP group and the normal group. The expression level of hub gene NELFB was consistent with bioinformatics (Figures 13A–C).

Generally speaking, Wilcoxon test combined with WGCNA methods were used to screen 91 DE-MRG related to the MRG score, in which AKT1 interacted with the most proteins in the PPI network. Next, NELFB, SFSWAP, and MAP3K3 were selected as the key genes with excellent diagnostic value for OP. These key genes was mainly enriched in the biological terms of cytoplasmic translation and Fc gamma R-mediated phagocytosis, especially, MAP3K3 involved in the activation and inhibition of various IPA Pathway, such as Cardiac Hypertrophy Signaling and GNRH Signaling. Moreover, there were considerable correlations between NELFB and iDCs (cor = −0.39), and Tfh levels (cor = 0.23). It is worth noting that the expression level of NELFB detected by RT-qPCR was consistent with the results of bioinformatics. Finally, the potential ceRNA network, TF binding sites, drugs targeting three key genes was predicted. For the clinical utilize, the nomogram constructed by converting the expression of three hub genes into a total score had a high prediction accuracy for osteoporosis (C-index = 0.886, 95% CI = 0.815-0.957; HL test *p* > 0.05).

## 4 Discussion

Basic research on mitophagy and osteoporosis has received considerable attention. This study evaluated the DEG between patients with OP and healthy cohorts and explored the key modules of MRG based on GSVA-WGCNA. The intersection of the 91 DE-MRG was analyzed using KEGG and GO. A PPI network was constructed, and the core target gene was AKT1. Three hub genes related to OP and mitophagy genes were identified by three machine learning methods, including NELFB, SFSWAP, and MAP3K3. ROC and nomogram models proved their diagnostic value. GO and KEGG analyses showed that the three hub genes were involved in the ribosome and cytoplasmic ribosome pathways. IPA showed that MAP3K3 was associated with six pathways, including GNRH Signaling. The ssGESA indicated that NELFB was highly correlated with iDCs (cor = −0.390, *p* < 0.001). The network based on hub genes showed that 3 TFs (TRIM22, CEBPG, and CEBPZ), 4 miRNAs (hsa-miR-132-3p, hsa-miR-182-5p, hsa-miR-212-3p, and hsa-miR-324-5p), and 4 drugs were involved in osteoporosis. Finally, the expression level of NELFB detected by RT-qPCR was basically consistent with bioinformatics.

We performed DE-MRG enrichment analysis on the selected samples, and the results showed that they were involved in many biological processes, including “I−kappaB kinase/NF−kappaB signal”, “regulation of podosome assembly”, and “podosome assembly”. The inhibition of NF−kappaB signal can affect the formation of osteoclasts and has a high correlation with osteoporosis. In addition, the most common molecular functions include ‘heterochromatin’ and ‘secretory granule lumen’. Aging of BMSC is the main cause of osteoporosis. Several studies have reported that related genes can promote or inhibit the occurrence of osteoporosis by regulating heterochromatin to interfere with MSC(51). For example, KDM4B inhibits the formation of H3K9me3 (heterochromatin) lesions to reduce MSC self-renewal and affect osteoporosis (Deng et al., 2021), whereas sIRT3 inhibits the generation of heterochromatin through the vulcanization of hydrogen sulfide, thereby inhibiting the senescence of BMSC and providing a new target for the treatment of osteoporosis (Liu et al., 2023). Secretory granules indirectly affect blood glucose levels by regulating insulin secretion, and diabetes is a clinical risk factor for OP.

The central node of the PPI molecular network is AKT1, which interacts with and performs molecular docking between small molecules and encoded proteins. AKT1 is a serine/threonine protein kinase known as Akt kinase (Akt1, Akt2 and Akt3) (Xia et al., 2020). Previous studies have shown that induced AKT1 expression promotes the proliferation of mesenchymal stem cells and ultimately inhibits their apoptosis, thereby alleviating osteoporosis (Wang et al., 2022). In addition, AKT1, as a core target gene, can regulate OP through the PI3K-Akt signaling pathway, which is manifested in the phosphorylation of AKT1 and the expression of PI3K to promote osteogenesis and regulate the progression of OP (Yang et al., 2019; Zhao et al., 2021; Liang et al., 2022). AKT has also been reported to affect osteoporosis by upregulating FOXO1 and enhancing the expression of bone turnover markers (ALP, OCN, Runx2, and Col1) and extracellular matrix mineralization (Cai et al., 2021). AKT1 plays a key role in OP by participating in multiple signaling pathways.

NELFB is a negative elongation factor B, also known as COBRA1, which is one of the four subunits of the NELF complex that controls the participation of RNA polymerase II (Pol II) in gene transcription and is essential for multiple biological processes. Osteoporosis is caused by an imbalance between osteoclasts and osteoblasts and occurs near hematopoietic cells in the bone marrow. Osteoblasts are the primary cells involved in bone formation and play a central role in hematopoiesis (Salamanna et al., 2020; Kim et al., 2021). Researchers have found that the absence of NELFB can induce excessive progenitor cell development during primitive and final hematopoiesis. Hematopoietic progenitor cells can bind hematopoietic growth factors to promote interactions between osteoblasts and hematopoietic stem cells (HSC), thereby affecting the number and activity of osteoblasts (Huang et al., 2022). Therefore, NELFB and osteoblasts appear to be associated with osteoporosis via hematopoietic progenitor cells (Calvi et al., 2003; Kim et al., 2011). It has been reported that NELFB has a synergistic effect with TCF1 in T cell response to cancer, while a non-coding RNA AK045490 inhibits osteogenic differentiation by down-regulating the expression of TCF1, LEF1 and Runx2. NELFB may affect osteogenic differentiation and bone formation by interfering with TCF1, thus providing a potential new drug target for osteoporosis (Li et al., 2019). In addition, NELFB can inhibit the transcription of estrogen receptor (ER) receptor gene, interfere with its binding to estrogen, inhibit the expression of osteoprotegerin (OPG), and promote the role of nuclear factor (NF)-κB ligand (RANKL), thereby promoting osteoclast formation and bone resorption activity, which is closely related to the pathogenesis of osteoporosis (Cheng CH. et al., 2022).

SFSWAP is a hypothetical splicing factor that encodes a protein containing an RS domain (SR-like). Proteins containing RS domains regulate RNA processing, including splicing, transcript extension, transcript stability, nuclear export, miRNA cleavage, and genomic stability (Moayedi et al., 2014). By studying the mutation process of the Notch signaling pathway, Moayedi et al. found that the SfSWAP gene has a synergistic relationship with the sub-allele of the Notch ligand Jagged1 (Jag1), which can play the same role in the same genetic pathway and interfere with other genes of Notch signaling (Moayedi et al., 2014). We speculate that SfSWAP is associated with Notch signaling pathway and Jag1 gene.

JAG1 is a traditional osteoporosis gene, which was confirmed using the cFDR method in a study by Hu et al. (2018). Through a large genome-wide association study and a follow-up replication study, Gong et al. identified JAG1 as a candidate gene for BMD regulation in different races and as a potential key factor in the pathogenesis of fractures (Kung et al., 2010). Researchers signaling has been reported to strictly control mammalian cell fate determination during embryogenesis and adulthood, which is essential for skeletal development and activity of skeletal cells (Zanotti and Canalis, 2013). Disorders in Notch signaling are related to human diseases that affect bones. Wang et al. identified JAG1 and NOTCH1 as L-R genes with ossification-related functions using single-cell RNA sequencing, which further confirmed the inevitable relationship between the two and osteoporosis (Wang S. et al., 2023). Through animal experiments, Hui et al. found that the targeted inhibition of JAG1/NOTCH1 signal transduction can promote poor osteogenic differentiation of bone marrow mesenchymal stem cells (Han et al., 2022). These studies reflect the inevitable connection between these two factors and osteoporosis. In addition, considering that both Notch pathway and Jagged1 gene are associated with SfSWAP, this may indicate the potential role of SFSWAP in the development of osteoporosis.

MAP3K3, also known as mitogen-activated protein kinase kinase kinase kinase kinase kinase 3, is a member of the serine/threonine protein kinase family that is generally expressed and acts as an oncogene (Zhang Y. et al., 2019). Many studies have reported that MAP3K3 is highly correlated with the progression and treatment of nasopharyngeal carcinoma (Yin et al., 2019), ovarian cancer (Zhang Y. et al., 2019), lung cancer (He et al., 2015), and breast cancer (Fan et al., 2014). This is a universally expressed cancer-related gene. Another study has reported that MAP3K3 may be associated with cerebral cavernous malformations. It are believed that the mutation of MAP3K3 can define a subclass of cerebral cavernous malformations (Weng et al., 2021). In addition, He et al. found that six genes, including MAP3K3, may play an important role in the regulation of BMD in women by analyzing the association between multiple gene expression profiles and bone mineral density variation. This study also provides new therapeutic targets for the treatment of osteoporosis (He et al., 2016). However, there is no direct evidence of a link between MAP3K3 and OP. Perhaps it can be used as an object to study the molecular mechanism of OP in the future.

In addition, GSEA enrichment of the hub genes revealed that all three genes were involved in the ribosome and cytoplasmic ribosome pathways. Studies have found that endoplasmic reticulum stress is significantly correlated with osteoblast and osteoclast differentiation and osteoclast formation during osteoporosis progression. During this process, the number of ribosomes in the endoplasmic reticulum membrane decreases significantly (Li et al., 2017). This indicates that ribosomes are associated with the progression of osteoporosis. In addition, it has been reported that the pathogenesis of osteoporosis in the elderly may be related to ribosome-related genes and pathways (Wang C. et al., 2023). These results suggest that ribosomes and cytoplasmic ribosomes play key roles in osteoporosis.

The use of multiple drugs, including gonadotropin-releasing hormone (GnRH) agonists and aromatase inhibitors, increases the risk of fractures (Krishnan and Muthusami, 2017). Studies have reported that long-term treatment with gonadotropin-releasing hormone (GnRH) agonists induces competitive and reversible GnRH receptor blockade, thereby inhibiting the release of gonadotropins and sex hormones. A reduction in sex hormones can lead to a variety of adverse reactions, including accelerated bone loss, which positively promotes the occurrence of OP (Mohamad et al., 2021). This suggests that GnRH Signaling may be associated with osteoporosis. Natural Killer Cell Signaling has also been speculated to be related to OP. Osteoporosis is the result of a ‘bone remodeling’ imbalance, and there is a close relationship between the immune system, bone physiology, and pathology. Srivastava et al. developed the term ‘immune osteoporosis’ to emphasize the role of immune cells in osteoporosis pathology (Srivastava et al., 2018). Saxena et al. have reported that natural killer cells belonging to the lymphoid lineage have congenital characteristics and play a role in osteoporosis. There are also literatures that have concluded through bioinformatics methods that there are significant differences in natural killer cells between osteoporosis patients and non-OP patients (Saxena et al., 2021). This is consistent with our findings. GPR48, a member of the G protein-coupled receptor (GPCR) superfamily, has been reported to inhibit osteoclast differentiation by antagonizing the interaction between RANK and its ligand-RANKL, thereby interfering with the OP process (Filipowska et al., 2022). In addition, GPR125 positively regulates osteoclasts through RANKL-stimulated MAPK and AKT-NF-κB signaling pathways to participate in the treatment of osteoporosis (Tang et al., 2022). In conclusion, G-Protein Coupled receptor Signaling is highly correlated with the development, diagnosis, and treatment of OP. There are few reports on the relationship between the other three inhibited pathways and OP, and further research is required.

iDCs showed the highest correlation with key genes among the four immune-related genes based on ssGSEA. Studies have shown that dendritic cells (DCs) are effective antigen-presenting cells that are widely distributed in the bone immune and/or mucosal mesenchymal interface and can affect OP by activating RANKL-nuclear factor kB receptor activator or RANK-osteoprotegerin (Liu and Teng, 2023). In addition, we found a positive correlation between NELFB and Tfh levels (*p* < 0.05). However, there are few reports in this field, and further studies are required to confirm this.

Both mRNA and miRNAs are indispensable components of ceRNA regulatory networks. It is undeniable that the associated miRNAs are equally important (Xu et al., 2016). Identifying the relationship between miRNAs and diseases not only improves the understanding of molecular mechanisms and disease pathogenesis, but also facilitates clinical diagnosis and treatment (Zhang L. et al., 2019). In our study, four miRNAs, namely hsa-miR-132-3p, hsa-miR-182-5p, hsa-miR-212-3p, and hsa-miR-324-5p, were identified in the circRNA-miRNA-TF mRNA regulatory network. To date, no study has directly explored the roles of these four miRNAs in osteoporosis. However, our data further support the possibility that these four miRNAs are important regulators of osteoporosis development. Nevertheless, this conjecture and its precise mechanism need to be further studied.

A transcription factor (TF), also known as gene promoter, is a protein that can bind to gene-specific sequences to mediate gene transcription and expression (Sebastian and Contreras-Moreira, 2013). Various transcription factors play regulatory roles in OP pathogenesis (Liu et al., 2019). In this study, TF-mRNA relationship pairs, including SFSWAP and MAP3K3, were obtained. The transcription factor TRIM22 is related to both genes. Many studies have shown that the absence of TRIM22 can affect the PI3K/Akt/mTOR pathway, and studies have reported that the latter has a potential relationship with OP progression (Li et al., 2018). In addition, Jiang et al. reported that the expression of the CCAAT/enhancer-binding protein (CEBP) homologous protein can promote BMP4-induced osteogenesis of MSCs *in vitro* and *in vivo*. Transcription factors CEBPG and CEBPZ may have the same effect (Jiang et al., 2018). The TF-mRNA relationship that we studied may provide strong evidence for verifying the potential relationship between hub genes and OP.

CTD is a powerful public database and its significance lies in increasing our understanding of how environmental exposure affects human health (Davis et al., 2021). We used key genes combined with the CTD database to predict potential drugs for OP treatment. The results showed that vinclozolin and bisphenol A were simultaneously associated with SFSWAP and MAP3K3, and benzo (a) pyrene and Valproic Acid were associated with both SFSWAP and NELFB. Studies have reported that some drugs may lead to osteoporosis. For example, bisphenol A inhibits osteoblast differentiation and bone formation by activating RORα, leading to the formation of osteoporosis (Maduranga et al., 2022). Previous studies have shown that bisphenol A is associated with a high prevalence of lumbar osteopenia and osteoporosis in postmenopausal women (Wang N. et al., 2020). Valproic Acid activates the Notch signaling pathway and has a positive effect on bone defect repair. A previous study reported that the Notch signaling pathway is closely related to osteoporosis (Tao et al., 2021). There is no evidence in previous reports that other drugs, such as vinclozolin and benzo (a) pyrene, play a role in OP; therefore, our study first speculated that vinclozolin and benzo (a) pyrene may have a therapeutic effect on OP, which needs to be verified by further related experiments.

We note that two recent articles are similar to our research, and we analyze the similarities and differences between them. These articles are roughly the same as our research in the overall research method, and the subjects of the study are osteoporosis. But the biggest difference is the choice of genes. Song Hao et al.selected immune-related genes. Because bone and immune system have a common developmental niche, the development and remodeling of bone are affected by the immune system. Immune cells can participate in the pathogenesis of OP by producing pro-inflammatory mediators. Huang Xinzhou directly selected differentially expressed genes in osteoporosis as the research object. We selected mitophagy-related genes for analysis. Secondly, both articles use machine learning methods to identify genes. In the former study, LASSO and mSVM-RFE methods are selected. The latter was analyzed by LASSO and the Gaussian mixture model. In this study, three machine methods, Lasso, SVM-RFE and Boruta, were selected for screening. Therefore, although these two studies are similar to our research, the final screening genes and results are not the same. In summary, our study does not repeat the previous steps, and there is a lot of innovation.

The data in this study were all from the selected data set GSE56815, which was only produced by human peripheral blood monocytes (PBMC). A number of studies have found that PBMC are involved in the occurrence of OP (Liu et al., 2015; Xie et al., 2023). It can produce a variety of cytokines and growth factors that affect bone metabolism, such as macrophage colony stimulating factor, interleukin 1, IL-6 and transforming growth factor β (Fischer and Haffner-Luntzer, 2022; Xie et al., 2023). In addition, monocytes are also precursors of osteoclasts with bone resorption activity. Under certain conditions, osteoclasts are produced by monocytes *in vitro* (Sprangers et al., 2016). However, although monocytes are the premise of osteoclasts, bone marrow can include osteoblasts, osteoclasts and osteocytes. Studies have found that almost all pathways in PBMC overlap with pathways in bone marrow tissue (Xie et al., 2023). Therefore, peripheral blood mononuclear cells can only reflect the situation in the bone marrow to a certain extent, and the study of OP through PBMC is limited. This is one of the limitations of this study. In addition, other limitations that should be recognized include: the sample size of data from public databases is relatively small, which can easily lead to selection bias; the verification step only selected clinical trial verification, and the corresponding database was not selected for further verification, which reduced the credibility of the results.

Although several mitophagy-related genes were screened in this study, and the reliability of our analysis was verified from the side by biological analysis, several conclusions were also speculated, including that SfSWAP affected the progress of OP by association with Notch signaling pathway and Jag1 gene. The 4 miRNAs may be important regulators in the development of osteoporosis. Vinclozolin and Benzo (a) pyrene may have therapeutic effects on OP. However, the potential regulatory mechanism of mitophagy in the progression of OP has not been fully elucidated, and the conclusions we speculate have not been determined. In the future, more *in vivo* and *in vitro* experiments and biological analysis are needed to further verify.

## 5 Conclusion

In general, this study studied the association between mitophagy-related genes and OP through a number of machine learning and biological analysis, and screened three hub genes, NELFB, SFSWAP and MAP3K3, which can be used as potential biomarkers for the diagnosis, mechanism research and treatment of osteoporosis. In addition, our study supports a more in-depth study of the mechanism of action of these three mitophagy-related genes in OP.

## Data Availability

The original contributions presented in the study are included in the article/Supplementary Material, further inquiries can be directed to the corresponding authors.

## Supplementary material

SUPPLEMENTARY TABLE S1 Lists for the differentially expressed genes between OP and control samples in GSE56815 datasets.

SUPPLEMENTARY TABLE S2 Results for the GSEA enrichment analysis of hub gene.

SUPPLEMENTARY TABLE S3 Lists for the differentially expressed immune-related gene sets between OP and control samples in GSE56815 datasets.

SUPPLEMENTARY TABLE S4 Results for the statistic analysis of spearman correlation analysis of differentially expressed immune-related gene sets (p value).
